# Ten Genome Sequences of Human and Livestock Isolates of *Bacillus anthracis* from the Country of Georgia

**DOI:** 10.1128/genomeA.00256-17

**Published:** 2017-05-11

**Authors:** Ekaterine Khmaladze, Giorgi Dzavashvili, Gvantsa Chanturia, Mikeljon P. Nikolich, Patrick S. G. Chain, Shannon L. Johnson, Paata Imnadze

**Affiliations:** a National Center for Disease Control & Public Health (NCDC), Tbilisi, Georgia; b Ivane Javakhishvili Tbilisi State University, Tbilisi, Georgia; c Georgian American University, Tbilisi, Georgia; d Los Alamos National Laboratory, Los Alamos, New Mexico, USA; e U.S. Army Medical Research Unit–Georgia, Tbilisi, Georgia; f Walter Reed Army Institute of Research, Silver Spring, Maryland, USA

## Abstract

*Bacillus anthracis* causes the acute fatal disease anthrax, is a proven biological weapon, and is endemic in Georgia, where human and animal cases are reported annually. Here, we present whole-genome sequences of 10 historical *B. anthracis* strains from Georgia.

## GENOME ANNOUNCEMENT

*Bacillus anthracis* causes cutaneous anthrax in humans and animals in Georgia and displays subspecies-specific differences in virulence, geographic distribution, and genetic diversity (1–3). Different molecular genotyping tools such as multiple-locus variable-number tandem repeat analysis (MLVA) and canonical single nucleotide polymorphisms (canonical SNPs) are used for genetic characterization of this organism (4, 5). In Georgia as well as other locations, SNPs are routinely used to subtype *B. anthracis* isolates and place them into a global phylogeographic context. We found two geographically distinct and relatively distant populations of *B. anthracis* that belong to different genetic groups defined by canonical SNPs (6). Five sequenced Georgian *B. anthracis* isolates belonged to the A.Br.013/015 clade and five to the A.Br.008/009 (Transeurasia) clade.

DNA fragment libraries were generated from genomic DNA according to the Illumina next-generation sequencing sample preparation method. *B. anthracis* DNAs were shredded by nebulization. The final size with an average of ca. 450 bp of the prepared libraries was determined by an Agilent 2100 bioanalyzer. Sequencing was performed using an Illumina 300 cycle sequencing kit on the MiSeq platform at NCDC Lugar Center in Tbilisi, Georgia. Obtained raw data of 150-bp length reads were analyzed using EDGE Bioinformatics (7). We assembled each draft genome using IDBA (7) in EDGE after quality trimming (standard parameters). Assemblies were aligned to the closest SNP subclade reference genomes, Ames ancestor and Sterne, for *B. anthracis*.

The draft genomes were annotated by utilizing the NCBI prokaryotic genomes automatic annotation pipeline (8) (PGAAP revision 3.3; http://www.ncbi.nlm.nih.gov/genomes/static/Pipeline.html).

### Accession number(s).

The whole-genome sequences for *B. anthracis* are available through GenBank under BioProject PRJNA336484 with the accession numbers listed in Table 1.

**TABLE 1:** Strain identifying information and basic statistics on assemblies and annotations

Strain ID	Yr of collection	Source of isolation	GenBank accession no.	No. of *de novo* contigs	Coverage depth (fold)	Contig *N*_50_ (bp)	No. of CDSs^a^
Ba-1802/12-Geo	2012	Patient ulcer	MVKJ00000000	78	119.35	365,205	5,722
Ba-1897/12-Geo	2012	Beef	MVKH00000000	88	132.10	413,677	5,723
Ba-8776/92-Geo	1992	Patient ulcer	MVKI00000000	81	141.80	331,561	5,719
Ba-9065/08-Geo	2008	Patient ulcer	MVKG00000000	70	209.11	331,561	5,724
Ba-9108/08-Geo	2008	Patient ulcer	MVKF00000000	95	157.55	432,600	5,727
Ba-7673/89-Geo	1989	Soil	MVKE00000000	86	211.16	868,539	5,618
Ba-8782/92-Geo	1992	Sheep skin	MVKD00000000	84	98.30	287,604	5,618
Ba-8784/92-Geo	1992	Beef	MVKC00000000	72	195.48	266,373	5,618
Ba-8785/92-Geo	1992	Patient ulcer	MVKB00000000	70	154.38	313,727	5,616
Ba-8884/94-Geo	1994	Patient ulcer	MVIR00000000	69	168.97	320,727	5,616

a CDSs, coding sequences.
